# Purification and Characterization of an Antibacterial Substance from *Aerococcus urinaeequi* Strain HS36

**DOI:** 10.4014/jmb.1910.10015

**Published:** 2019-11-04

**Authors:** Ho Sun Sung, Youl-Lae Jo

**Affiliations:** Department of Applied Microbiology and Biotechnology, Yeungnam University, Gyeongsan 38541, Republic of Korea

**Keywords:** *Aerococcus urinaeequi*, antibacterial substance, aquaculture, bacteriocin, *Vibrio* (*Listonella*) anguillarum

## Abstract

A bacterial strain inhibiting the growth of *Vibrio anguillarum*, the causative agent of vibriosis, was isolated from fish intestines. The isolated strain HS36 was identified as *Aerococcus urinaeequi* based on the characteristics of the genus according to *Bergey’s Manual of Systematic Bacteriology* and by 16S rRNA sequencing. The growth rate and antibacterial activity of strain HS36 in shaking culture were higher than those in static culture, while the optimal pH and temperature for antibacterial activity were 7.0 and 30°C, respectively. The active antibacterial substance was purified from a culture broth of *A. urinaeequi* HS36 by Sephadex G-75 gel chromatography, Sephadex G-25 gel chromatography, and reverse-phase high-performance liquid chromatography. Its molecular weight, as estimated by Tricine SDS-polyacrylamide gel electrophoresis, was approximately 1,000 Da. The antibacterial substance produced by strain HS36 was stable after incubation for 1 h at 100°C. Although its antibacterial activity was optimal at pH 6-8, activity was retained at a pH range from 2 to 11. The purified antibacterial substance was inactivated by proteinase K, papain, and β-amylase treatment. The newly purified antibacterial substance, classified as a class II bacteriocin, inhibited the growth of *Klebsiella pneumoniae*, *Salmonella enterica*, and *Vibrio alginolyticus*.

## Introduction

Fish diseases not only cause rising production costs in the short term, but may also hinder the development of the aquaculture industry in the long run. Although fish mortality may occur due to natural selection, viral, bacterial, and parasitic diseases are the most common cause of death [1]. Among the latter, bacterial diseases lead to great economic losses in aquaculture farms because they occur at high frequencies and spread easily among fish populations [2]. This is because breeding conditions that are optimal for fish growth are also optimal for bacteria and highly contagious bacterial diseases. Fish bacterial diseases are mainly caused by *Aeromonas hydrophila*, *Edwardsiella tarda*, *Streptococcus parauberis*, and *Vibrio anguillarum*. *V. anguillarum* is especially important because it is very abundant in marine environments and is pathogenic to at least 90 different species of fish, crustaceans, and bivalves [3, 4]. In addition, pathogen virulence usually increases under fish farming conditions because fish populations show diminished immune systems when bred at high densities [5, 6].

Disease control in cultured fish is generally performed through antibiotic treatment when primary prevention is insufficient and there is a disease outbreak. Antibiotics were first approved by the USFDA in 1951 and have been used since then together with animal feed, to significantly reduce the number of deaths from bacterial infections. However, the development of antibiotic-resistant pathogenic strains due to overutilization of antibiotics has become a serious problem [7, 8].

Several studies have focused on the development of probiotics as an alternative to enhance fish immunity [9-11]. For example, Cha *et al*. [12] reported that when food for flounders was supplemented with *Bacillus* spp., the fish showed increased resistance to disease. However, the activity of microorganisms is easily inhibited by gastric acid or other digestive fluids, preventing their settlement in the gastrointestinal tract; thus, using one specific beneficial microorganism in a fish population does not warrant its success in another. Therefore, the use of antibacterial proteins, like bacteriocins which are produced by probiotics, is an alternative to both probiotics and antibiotics.

Bacteriocins are ribosomally synthesized antibacterial peptides or proteins produced by bacteria and archaea, which act as competitive growth inhibitors for similar or closely related strains [13, 14].

They are also produced by many microorganisms isolated from fermented foods such as dairy products and kimchi. Unlike antibiotics, bacteriocins are proteins that can easily be degraded by proteolytic enzymes, they are non-toxic to humans, and leave no residues in the body. Therefore, they can be safely used as natural food preservatives or medicines [15]. However, most bacteriocins have a relatively narrow spectrum of antibacterial effects. Furthermore, studies on bacteriocins have focused on their effect on gram-positive pathogens, whereas studies on gram-negative bacteria are still insufficient.

In this study, we isolated and identified a bacterial strain, *A. urinaeequi* HS36, that produces an antibacterial substance against the gram-negative pathogen *V. anguillarum*. We also purified and characterized this new antibacterial substance and found that it is a thermostable bacteriocin active against gram-negative bacteria.

## Materials and Methods

### Bacterial Indicator Strain and Media

*V. anguillarum* (also known as *Listonella anguillarum*) ATCC 19264 was used as the indicator strain. It was grown in LB medium at 25°C.

### Isolation of a Strain with Antibacterial Activity against *V. anguillarum*

A bacterial strain capable of inhibiting the growth of *V. anguillarum* was isolated from the intestines of cod collected from a local market in Daegu, Korea. Briefly, collected samples were suspended in 10 ml of nine salt solution (NSS), serially diluted, and cultured on thiosulfate-citrate-bile-sucrose agar (TCBS; Difco, USA) at 25°C for 24 h. Isolated colonies were selected with a sterile sharp-ended toothpick and inoculated on an LB agar plate overlaid with *V. anguillarum*. After incubation at 25°C for 24 h, the antibacterial activity of the colonies was determined by the presence of growth inhibition (clear) zones surrounding them.

### Identification of the Isolated Strain

The strain with the highest antibacterial activity against *V. anguillarum*, strain HS36, was characterized and identified according to *Bergey’s Manual of Systematic Bacteriology* [16] based on morphological, physiological, and biochemical characteristics. Strains were also identified using 16S rRNA sequence analysis; to this end, chromosomal DNA from the isolated strain was extracted using purification beads (Solgent, Korea). Next, this DNA was used as a template to amplify the 16S rRNA gene through polymerase chain reaction (PCR) using universal primers (27F: 5’-AGA GTT TGA TCC TGG CTC AG-3’ and 1492R: 5’-GGT TAC CTT GTT ACG ACT T-3’). PCR products were purified using a PCR purification kit (Solgent), and the nucleotide sequences were determined by Sanger sequencing on an ABI 3730XL DNA sequencer (Applied Biosystems, USA). Homology searches against NCBI databases were conducted using the BLASTN program. Phylogenetic analysis was performed using Mega4 (MEGA Software, USA) both with the neighbor-joining method and an equal input model, which allow for variations in the substitution rates at one site and among all sites.

### Growth and Antibacterial Activity of Isolated Strain

Strain HS36 was cultivated in LB broth at 30°C; its growth and antibacterial substance production rates were measured using a UV spectrometer and by the paper disc method, respectively, by sampling every hour for 24 h.

### Determination of Antibacterial Activity

Strain HS36 was cultivated in MRS/NSS broth at 30°C for 18 h and centrifuged at 10,000 ×g for 15 min. Then, the supernatant was filtered through a 0.45-μm membrane filter (Advantec MFS, Inc., Japan) and freeze-dried. Freeze-dried supernatant (5-fold concentrated) was resuspended in 20 mM potassium phosphate buffer (pH 7.0) and antibacterial activity was determined by the paper disc method (8 mm diameter) on LB plates overlaid with *V. anguillarum* at 25°C for 24 h. The diameters of the growth inhibition areas (including the filter paper) were measured using a Vernier caliper.

### Purification of the Antibacterial Substance

Purification of the antibacterial substance produced by strain HS36 from a culture broth was performed in three steps: by Sephadex G-75 gel filtration, Sephadex G-25 gel filtration, and reverse-phase high-performance liquid chromatography (RP-HPLC). Briefly, the culture broth was centrifuged at 10,000 ×g for 15 min, then the supernatant was loaded onto a Sephadex G-75 gel filtration system (2.0 × 75 cm) equilibrated with 20 mM potassium phosphate buffer (pH 7.0). The column was eluted at a flow rate of 0.14 ml/min, and the eluted antibacterial substance fraction was applied to a Sephadex G-25 column (2.0 × 75 cm). The column was eluted with the same buffer at a flow rate of 0.12 ml/min. Finally, fractions were separated on an RP-HPLC system (Agilent Eclipsed XDB-C18). The sample was eluted at a flow rate of 0.2 ml/min with 20% methanol as the solvent. Eluates were monitored by measuring the UV absorbance at 220 nm. The antibacterial activity of each fraction was determined using the paper disc method and the active fraction was freeze-dried.

The molecular weight of the purified antibacterial substance was determined by Tricine SDS-PAGE (16% acrylamide separating gel and 4% acrylamide stacking gel). Biological activity against *V. anguillarum* was confirmed by the gel overlay test at 30°C.

The arbitrary units (AU) were determined by the paper disc method (8 mm diameter) using two-fold serial dilutions of the antibacterial substance. The activity of the antibacterial substance was defined as the reciprocal of the dilution after the lowest serial dilution that inhibited growth.

### Characterization of the Antibacterial Substance

First, the heat stability of the antibacterial substance was monitored. Samples from the purified fraction were heated at temperatures ranging from 10–100°C for 3 h; samples were also incubated at 121°C for 15 min, 30 min, and 1 h. Second, the stability of the antibacterial substance at various pH values was measured after mixing it with buffers of different pH values: 100 mM citric acid buffer at pH 2.0, 3.0, 4.0, and 5.0; 100 mM potassium phosphate buffer at pH 6.0 and 7.0; 100 mM Tris-HCl buffer at pH 8.0 and 9.0; or 100 mM NaHCO_3_-NaOH buffer at pH 10.0 and 11.0 at 4°C for 12 h. Third, the stability of the antibacterial substance in the presence of various metal ions and inhibitors (100 mM each) was measured after mixing and incubating at 4°C for 12 h. Fourth, the effect of enzymes on the activity of the antibacterial substance activity was determined after incubation with α-amylase, β-amylase, chymotrypsin, lipase, lysing enzyme, lysozyme, papain, pepsin, pronase, protease, proteinase K, or trypsin (all enzymes at 2 mg/ml, Sigma-Aldrich) at optimal temperatures for 3 h. Then, the mixtures were heated to 100°C for 10 min to denature the enzymes. The residual activity of the antibacterial substance was measured using the paper disc method. Finally, the spectrum of antibacterial activity of the isolated substance against pathogenic microorganisms, such as gram-positive bacteria, gram-negative bacteria, mold, and yeast, was tested by the paper disk method: a clear inhibition zone at least 9 mm in diameter was scored as positive.

## Results and Discussion

### Isolation and Identification of Strain HS36

Bacteria were isolated from the intestines of cod obtained in the Daegu area of Korea. Among them, 7 isolates inhibited the growth of *V. anguillarum* ATCC19264, with strain HS36 possessing the highest antibacterial activity.

**Fig. 1:**
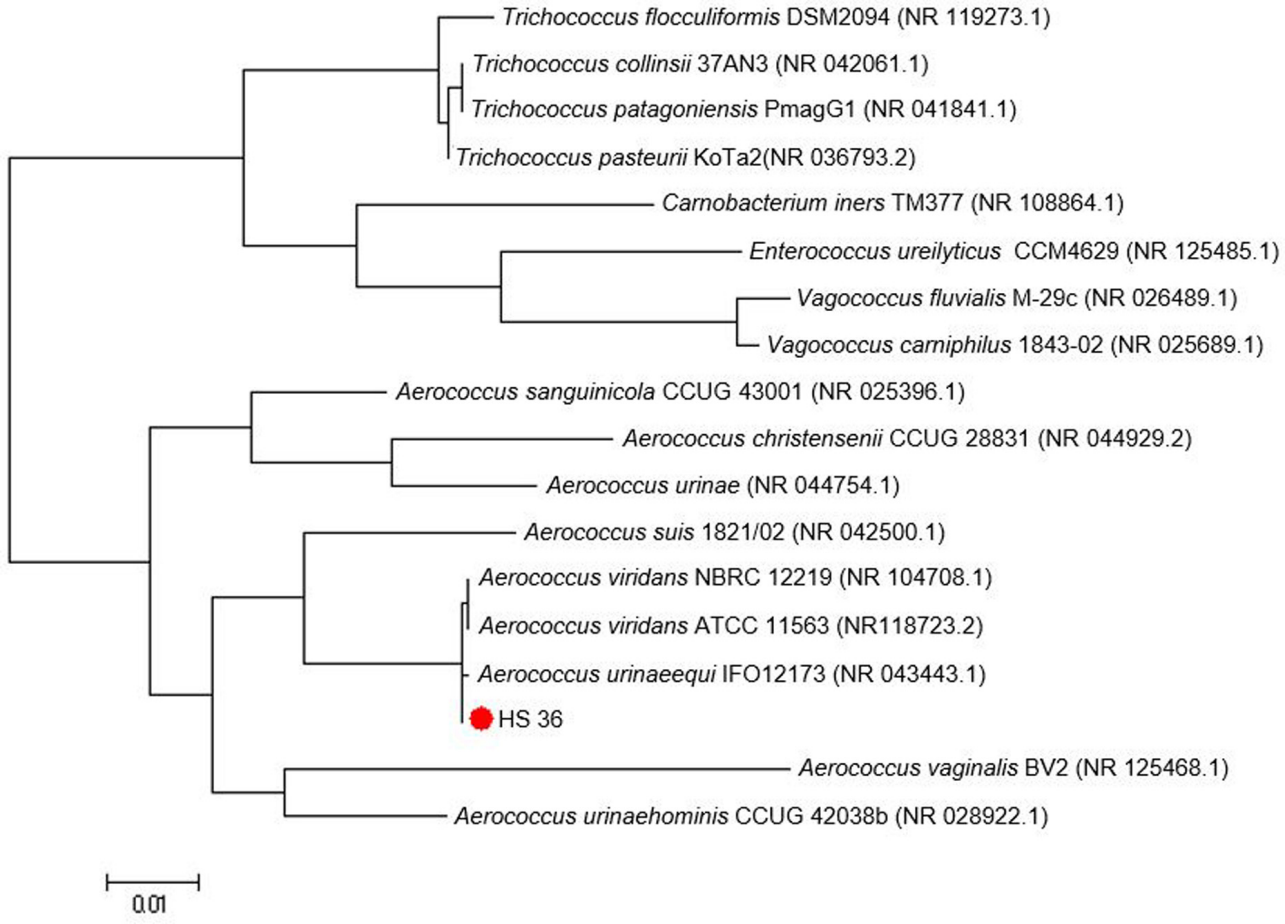
Phylogenetic position of strain HS36 among related *Aerococcus* spp. Phylogenetic tree that was generated using the neighbor-joining method with Mega4 software. Bar, 0.005 substitutions/nucleotide.

According to *Bergey’s Manual of Systematic Bacteriology* and experimental results, strain HS36 is classified as a gram-positive coccus, shows positive for catalase and methyl red (MR) tests, and belongs to the genus *Aerococcus*. Moreover, the 16S rRNA sequence of strain HS36 showed high similarity (99%) to that of *A. urinaeequi* IFO12173. Thus, it was designated as *A. urinaeequi* HS36. The cluster formed by HS36 and *A. urinaeequi* IFO12173 was supported by high bootstrap values (Fig. 1).

Since many bacteriocins produced by gram-positive bacteria, especially by lactic acid bacteria (LAB), are biologically safe and many studies have been conducted in the food industry to use them as natural preservatives, active antibacterial compounds isolated from *Aerococcus* strains (which are LAB) have potential as feed additives [17, 18].

### Production and Purification of an Antibacterial Substance from *A. urinaeequi* HS36

**Fig. 2:**
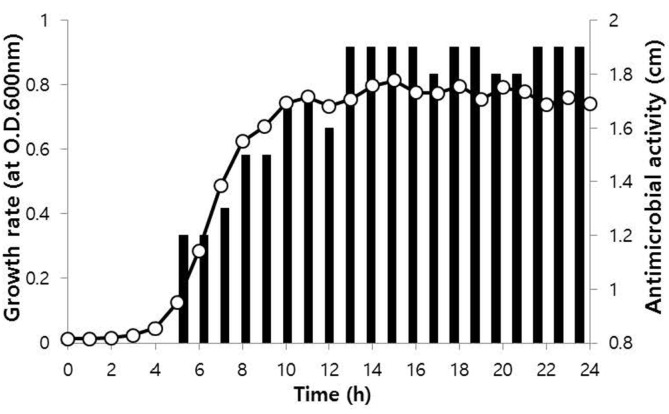
Cell growth curve and antibacterial activity of *Aerococcus urinaeequi* HS36. The isolated strain was inoculated in LB broth at 30°C. The growth and production of antibacterial substances were measured using a UV spectrometer and paper disc method every hour for 24 h. -○-, growth; ■ , clear zone size (cm).

Fig. 2 shows the growth of *A. urinaeequi* HS36 and its production rate of an antibacterial substance against *V. anguillarum*. Since the antibacterial substance was first produced at the exponential growth phase (after 5 h of incubation), it was suggested that it was not a secondary metabolite, such as an antibiotic, but rather a primary metabolite, such as a bacteriocin [19, 20]. The highest antibacterial activity was recorded when bacterial growth reached the stationary phase (after 13 h of incubation).

**Fig. 3:**
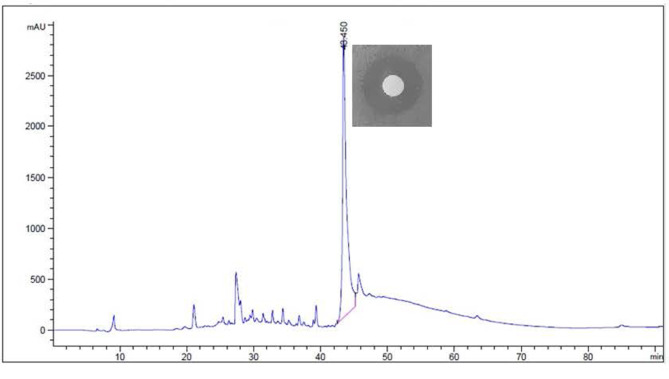
Reverse-Phase High-Performance Liquid Chromatography (RP-HPLC) purification of the antibacterial substance. Column size; 0.46 × 15 cm (5 μm), flow rate; 0.12 ml/min, mobile phase; 20% methanol. The last step of purification was performed by HPLC. The substance purified from C18 showed a single peak a retention time of 43.45 min. The active fraction containing the antibacterial substance was detected by determining the antibacterial activity against *V. anguillarum*.

After column purification and during RP-HPLC, the antibacterial substance extracted from the supernatant of a culture broth of *A. urinaeequi* HS36 showed a single peak at a retention time of 43.45 min. The corresponding fractions showed the antibacterial activity detected in the culture supernatant (Fig. 3). A summary on the purification of the antibacterial substance is shown in Table 1. The antibacterial substance extracted from *A. urinaeequi* HS36 was purified 1.4-fold, showed a specific activity of 2,037 AU/mg, and had a yield of 20%.

### Tricine SDS-PAGE Analysis of the Purified Antibacterial Substance

**Fig. 4:**
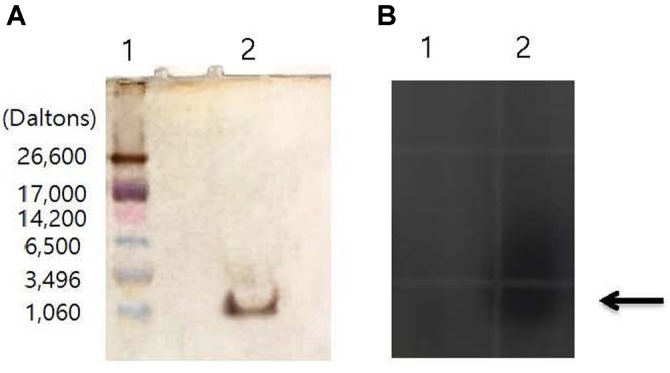
Molecular weight determination and confirmation of antibacterial activity by Tricine SDS-PAGE. (**A**) Gel with silver staining. (**B**) Overlaid gel with *Vibrio anguillarum*. lane 1, ultralow range molecular weight maker; lane 2, purified antibacterial substance by reverse-phase high-performance liquid chromatography.

The RP-HPLC fractions containing antibacterial activity were also analyzed by Tricine SDS-PAGE to monitor the purity of the purified antibacterial substance and to estimate its molecular weight. Tricine SDS-PAGE showed a single band of an approximate molecular weight of 1,000 Da (Fig. 4A). When a gel overlay test was performed at 30°C, the inhibitory region for *V. anguillarum* was observed at the same position as the band (Fig. 4B). An analysis of the composition of the first amino acids from the N-terminal domain of the peptide showed that they were of a hydrophobic nature (data not shown). As a small molecular weight thermostable peptide composed of hydrophobic amino acids, the isolated antibacterial compound was classified according to the classification system proposed by Klaenhammer [21] as a Class II bacteriocin. This group includes thermally stable bacteriocins with molecular weights below 12 kDa [22, 23].

### Characterization of the Isolated Antibacterial Substance

**Fig. 5:**
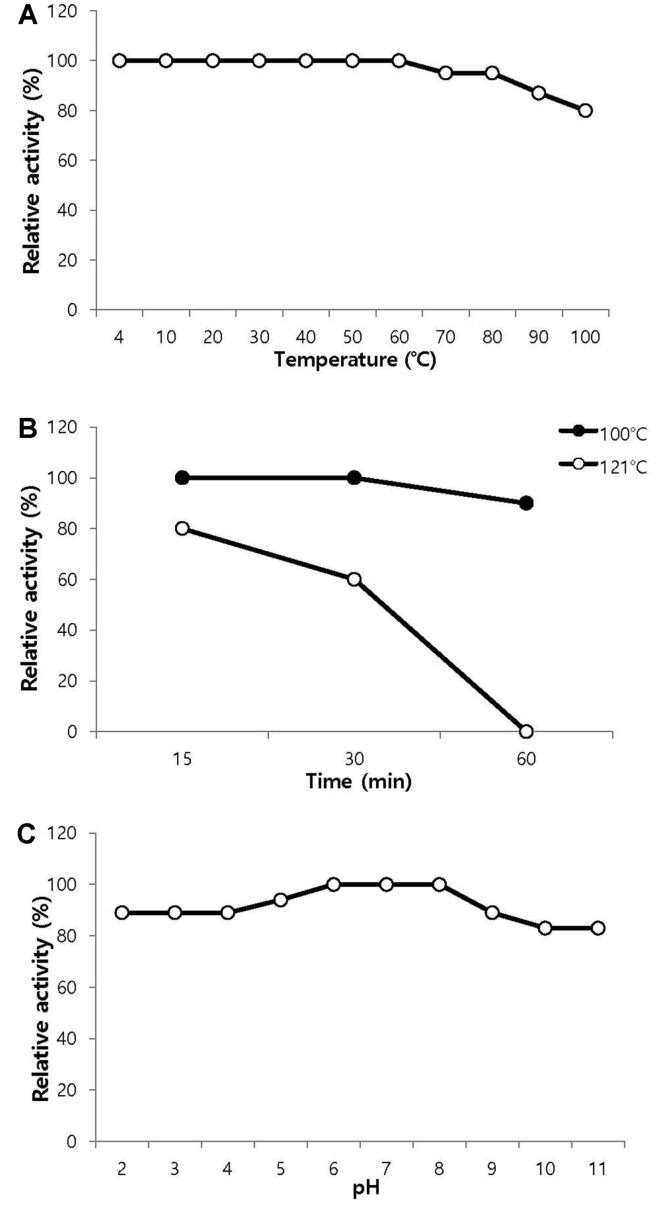
Effects of temperature and pH on the activity of the isolated antibacterial substance. (**A**) Effects of temperature. The purified antibacterial substance was heated at 10, 20, 30, 40, 50, 60, 70, 80, 90, and 100°C for 3 h. (**B**) Thermostability (Heat resistance). (**C**) Effect of pH. The stability of the antibacterial substance at various pH values was measured after mixing the antibacterial substance with buffers of different pH values (100 mM citric acid buffer [pH 2.0, 3.0, 4.0, and 5.0], 100 mM potassium phosphate buffer [pH 6.0 and 7.0], 100 mM Tris-HCl buffer [pH 8.0 and 9.0], or 100 mM NaHCO_3_-NaOH buffer [pH 10.0 and 11.0]) at 4°C for 12 h.

The antibacterial substance isolated from strain HS36 was remarkably heat-stable; 100% of its antimicrobial activity remained after incubation at 60°C for 3 h and 80%of its activity remained after 15 min at 121°C (Figs. 5A and 5B). The effect of pH on the antibacterial activity showed that the antibacterial substance retained 100% of its activity at pH 6–7 and more than 80% of its activity within a pH range from 2–11 (Fig. 5C).

The compound’s activity decreased approximately 11–17% after treatment with inhibitors, such as SDS and urea. It also showed a moderate resistance to various metal ions, such as AgNO_3_, C°Cl_2_, MgSO_4_, and MnSO_4_ (Table 2). Peptide molecules of a relatively small size, with an uncomplicated structure (without tertiary structures) are not expected to be significantly affected by temperature, pH, metal ions, or various inhibitors [24, 25].

According to a study by Huh and Hwang [26], there are non-peptidyl antibacterial molecules, such as benzoic acid and lactic acid, among many active substances secreted by LAB. However, the substance isolated from strain HS36 was assumed to be a peptide containing carbohydrate moieties, not only for the properties already mentioned, but also because it was inactivated by papain, proteinase K, and β-amylase (Table 2) [27]. In addition, an attempt to analyze the amino sequence of the N-terminal was made with the Edman degradation method. Although only the first four amino acids (NH_2_-FPPQ) were read and the sequence from the fifth was not identified due to any sugar moiety, the proteic nature of the compound was confirmed.

### Spectrum of Antibacterial Activity

The antibacterial substance produced by strain HS36 showed strong activity against *V. anguillarum*, *V. alginolyticus*, *Klebsiella pneumoniae*, and *Salmonella enterica* (Table 3). In addition, it showed antibacterial activity against several other gram-negative bacteria, but not against gram-positive bacteria. Thus, it can be concluded that this newly isolated Class II bacteriocin has a broad antibacterial spectrum, and it is effective for regulating gram-negative pathogenic bacteria.

Nisin, a bacteriocin typically produced by *Lactococcus lactis*, is representative of those antibacterial agents with broad-spectrum activity against gram-positive bacteria such as *Listeria monocytogenes*, *Staphylococcus aureus*, and *Bacillus cereus* [28]. Since most bacteriocins are known to inhibit bacterial growth by causing the collapse of the proton motive force [29], they have an inhibitory effect on gram-positive bacteria, but not on gram-negative bacteria since gram-negative bacteria have an outer membrane. According to Belfiore *et al*. [30], it is possible to extend the effect of bacteriocins to gram-negative bacteria using chelating agents such as EDTA. In addition, the overall antimicrobial activity of the purified substance increased antimicrobial activity when EDTA was added to the medium.

In this study, we isolated and identified potential antibacterial substance-producing microorganisms that could be commercialized and used to prevent economic losses in the aquaculture industry. Also, we purified and characterized a new antibacterial substance produced by *A. urinaeequi* HS36, which had not yet been reported to produce bacteriocins.

The purified antibacterial substance was a proteinaceous molecule that is remarkably stable against heat and has a molecular weight of about 1,000 Da.

These results suggest that the bacteriocin from *A. urinaeequi* HS36 may be effective for inhibiting the growth of gram-negative pathogenic bacteria in aquaculture.

## Figures and Tables

**Table 1:** Summary of the purification profile of an antibacterial substance from the culture supernatant of *Aerococcus urinaeequi* HS36.

Purification step	Total volume (ml)	Total protein (mg)	Activity (AU/ml)	^a^Total activity (AU)	^b^Specific activity (AU/mg)	^c^Fold	^d^Yield(%)
Crude extract	500	40.2	120	60000	1491	1	100
Sephadex G-75	100	10.2	480	48000	4720	3.2	80
Sephadex G-25	75	6.2	240	18000	2899	1.9	30
RP-HPLC	50	5.9	240	12000	2037	1.4	20

^a^Total activity (AU): represents Activity (AU/ml) × Volume (ml)

^b^Specific activity (AU/mg): represents total activity divided by protein concentration.

^c^Purification fold: represents specific activity of purified fraction divided by specific activity of crude extract Activity (AU/ml) × Volume (ml)

^d^Yield (%): represents (total activity of purified fraction divided by total activity of crude extract) × 100

**Table 2:** Effect of various enzymes, metal ions, and chemical inhibitors on the activity of the antibacterial substance from *Aerococcus urinaeequi* HS36.

	Residual activity (%)		Residual activity (%)
Enzyme		Metals ions	
α-amylase	80	AgNO₃	89
β-amylase	30	BaCl₂	94
Chymotrypsin	60	CaCl₂	105
Lipase	80	C°Cl₂	89
Lysing enzyme	100	CuSO₄	94
Lysozyme	90	FeSO₄	100
Papain	0	MgSO₄	83
Pepsin	70	MnSO₄	78
Pronase	70	SnCl₂	100
Protease	70	ZnSO₄	100
Proteinase K	0	Chemical inhibitors	
Trypsin	80	EDTA	110
		SDS	89
		Sodium azide	94
		Titon X-100	94
		Tween 80	94
		Urea	83
Control	100		

α-Amylase (endo-type enzyme), β-Amylase (exo-type enzyme): hydrolyzes the α-(1,4) glucan linkages in polysaccharides of three or more α-(1,4) linked Dglucose units; Chymotrypsin: selectively hydrolyzes peptide bonds on the Cterminal side of tyrosine, phenylalanine, tryptophan, and leucine; Lipases: hydrolysis of triacylglycerols into glycerol and free fatty acids; Lysing enzymes: hydrolyzes oligosaccharides to glucan; Lysozyme: hydrolyzes β(1 → 4) linkages between N-acetylmuramic acid and N-acetyl-D-glucosamine residues in peptidoglycan; Papain: cysteine protease; pepsin: hydrolyzes only peptide bonds, not amide or ester linkages; Pronase: hydrolyzes proteins down to single amino acids; Protease: enzyme used to break down proteins by hydrolyzing peptide bonds; Proteinase K: serine protease; Trypsin: cleaves peptides on the Cterminal side of lysine and arginine residues. EDTA: ethylene diamine tetra acetic acid; SDS: sodium dodecyl sulfate.

**Table 3:** Antibacterial activity of the compound isolated from *Aerococcus urinaeequi* HS36 against various bacteria.

Microorganism	Indicator species	Antimicrobial activity
Gram-positive		
bacteria	*Bacillus cereus* KCTC1012	-
	*Staphylococcu aureus* KCTC3658	-
	*Streptococcus salivarius* KCTC3744	-
	*Streptococcus mutans* KCTC5365	-
	*Streptococcus Sobrinus* KCTC3308	-
Gram-negative		
bacteria	*Edwardsiella tarda* KCTC12267	-
	*Enterobacter aerogenes ATCC13048*	+
	*Enterobacter cloacae* KCTC2361	+
	*Escherichia coli* KCTC2441	-
	*Klebsiella pneumoniae* KCTC2208	+++
	*Vibrio anguillarum* KCTC2711	+++
	*Salmonella enrica ATCC10727*	++
	*Vibrio alginolyticus* KCTC2928	+++
Mold	*Aspergillus niger KCTC6592*	-
	*Aspergillus flvus* KCTC6984	-
	*Botryotinia fuckeliana* KCTC6973	-
	*Penicillium rubrisclerotium* KCTC6773	-
Yeast	*Candida albicans* KCTC7270	-
	*Zygosaccharomyces roxii* KCTC7880	-

Activity was expressed as the diameter of inhibition zone against each sensitive indicator. Degree of clarity of clear zone by growth inhibition: -, ≤ 0.8 cm (negative); +, 0.9–1.1 cm (moderate inhibitory activity); ++, 1.1–1.3 cm (strong inhibitory activity): +++, > 1.3 cm (very strong inhibitory activity).
